# Supplementary figures and images for: Model-based prioritization for acquiring protection
Source: PLoS Comput Biol. 2022 Dec 19;18(12):e1010805. doi: 10.1371/journal.pcbi.1010805 (PMC9810162; doi:10.1371/journal.pcbi.1010805)

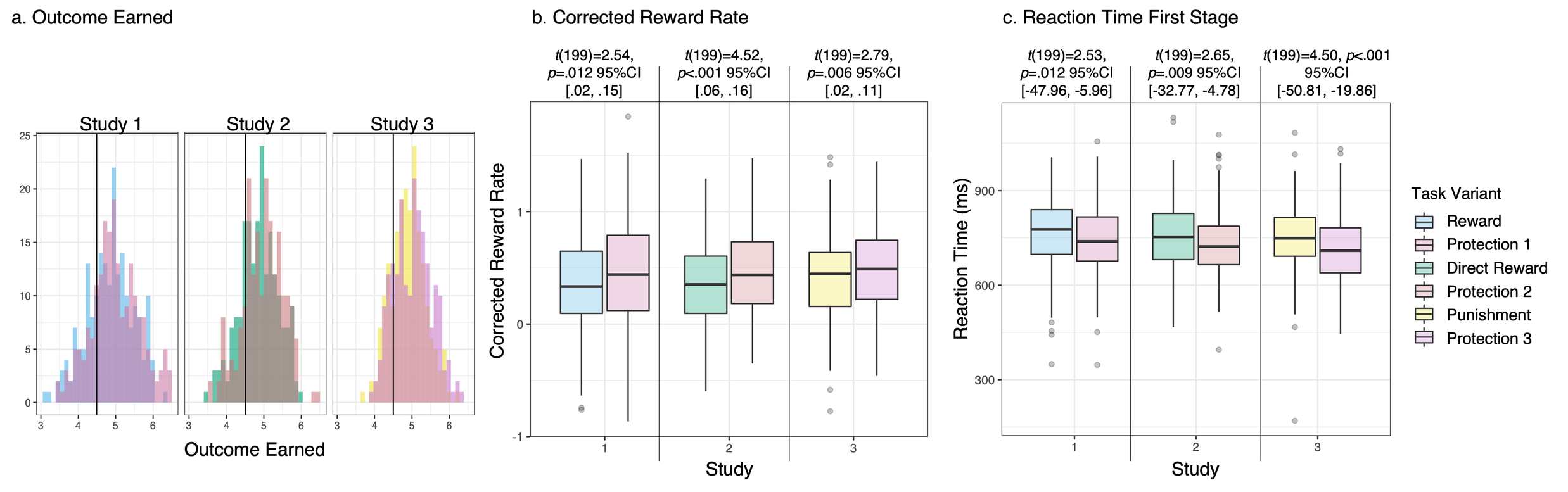

Supplement: S1 Fig — a. Histograms depicting the number of outcome units earned by study and task variant. Black lines indicate median available outcome for each study. b. Corrected reward rate boxplots by study and task variant. Corrected reward rate was significantly higher for the protection task variants compared to all other task variants. Corrected reward rate was calculated as the average outcome earned divided by average outcome available, which was determined by the randomly drifting outcome distributions generated for each subject. c. Reaction time (milliseconds, ms) boxplots by study and task variant. Subjects made first-stage decisions quicker for the protection task variants compared to all other task variants. Far right legend indicates task variants across all studies: Study 1 = Reward and Protection 1, Study 2 = Direct Reward and Protection 2, Study 3 = Punishment and Protection 3. (TIF) [file pcbi.1010805.s001.tif]

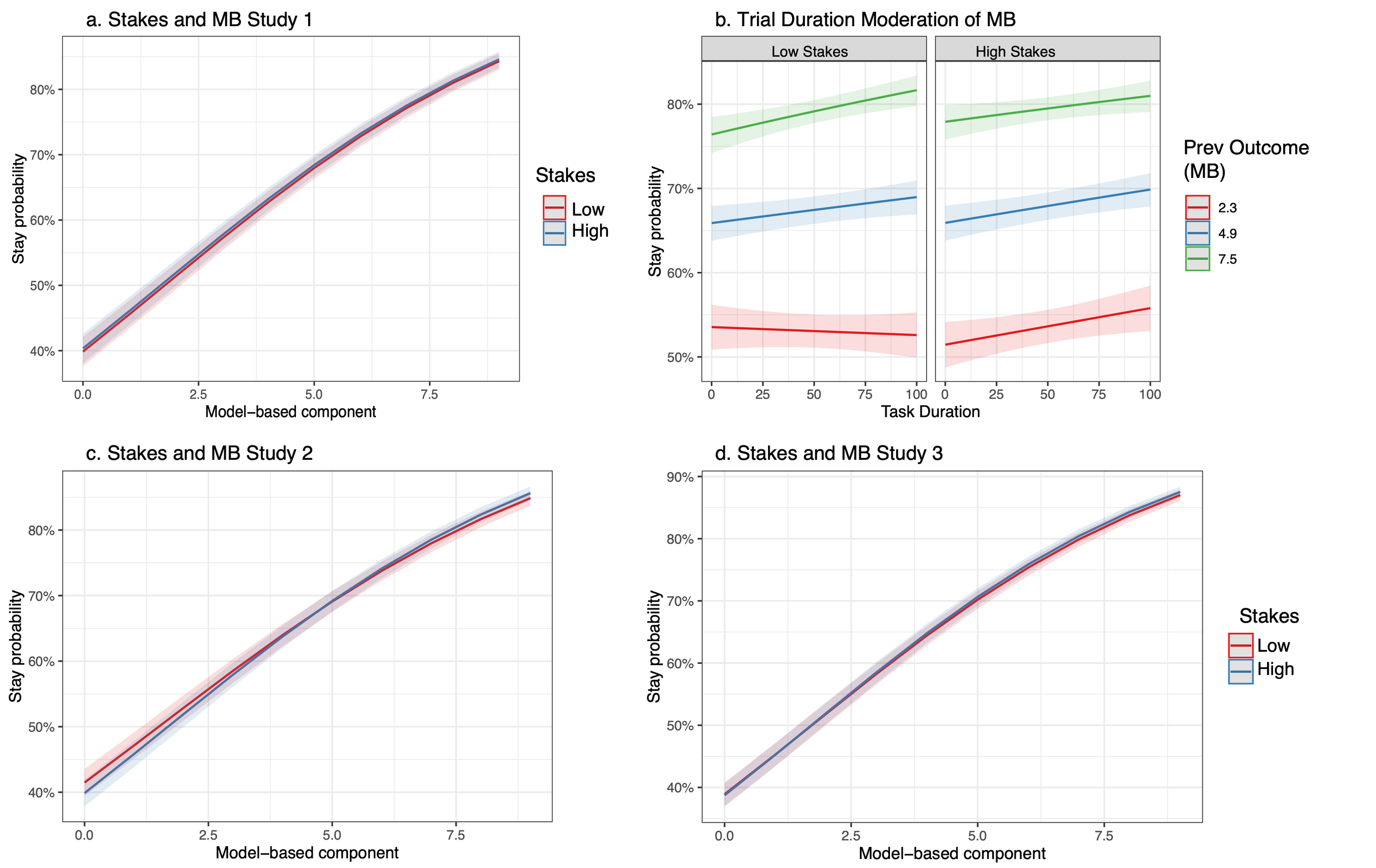

Supplement: S2 Fig — We assessed whether use of model-based control was affected by stakes by testing whether stakes moderated stay behavior in mixed model analyses. (a) Stakes did not significantly interact with either the model-based or model-free component across tasks in Study 1: MB Estimate = .0001, SE = .009, z = .02, p = .988, 95% CI [-.02, .02], τ00 = .48, R2 = .15; MF Estimate = -.02, SE = .02, z = .89, p = .375, 95% CI [-.02, .05], τ00 = .49, R2 = .17. (b) Task duration interacted with stakes and previous outcome, such that there was no effect of stakes at the start of the task but high-stakes trials had an increase in likelihood of stay behavior at the end of the task: Estimate = -.001, SE = .0003, z = -2.10, p = .036, 95% CI [-.001, -.00004], τ00 = .48, R2 = .16. (c) Study 2, which increased trials to 200 non-practice (compared with 100 non-practice in Study 1), which revealed a stakes effect that interacted with the model-based component: Estimate = .01, SE = .006, z = 2.20, p = .028, 95% CI [.002, .03], τ00 = .51, R2 = .16. This effect was driven by the direct reward variant: direct reward Estimate = .02, SE = .009, z = 2.68, p = .007, 95% CI [.006, .04], τ00 = .59, R2 = .17; protection Estimate = .004, SE = .009, z = .37, p = .711, 95% CI [-.02, .02], τ00 = .66, R2 = .21. No significant interaction was present for the model-free component: Estimate = -.007, SE = .01, z = -.53, p = .595, 95% CI [-.03, .02], τ00 = .51, R2 = .17. (d) In Study 3, the stakes effect was not significant with respect to either model-based or model-free component: MB Estimate = .006, SE = .007, z = .88, p = .381, 95% CI [-.01, .02], τ00 = .46, R2 = .17; MF Estimate = .02, SE = .01, z = 1.87, p = .062, 95% CI [-.001, .05], τ00 = .46, R2 = .18. (TIF) [file pcbi.1010805.s002.tif]

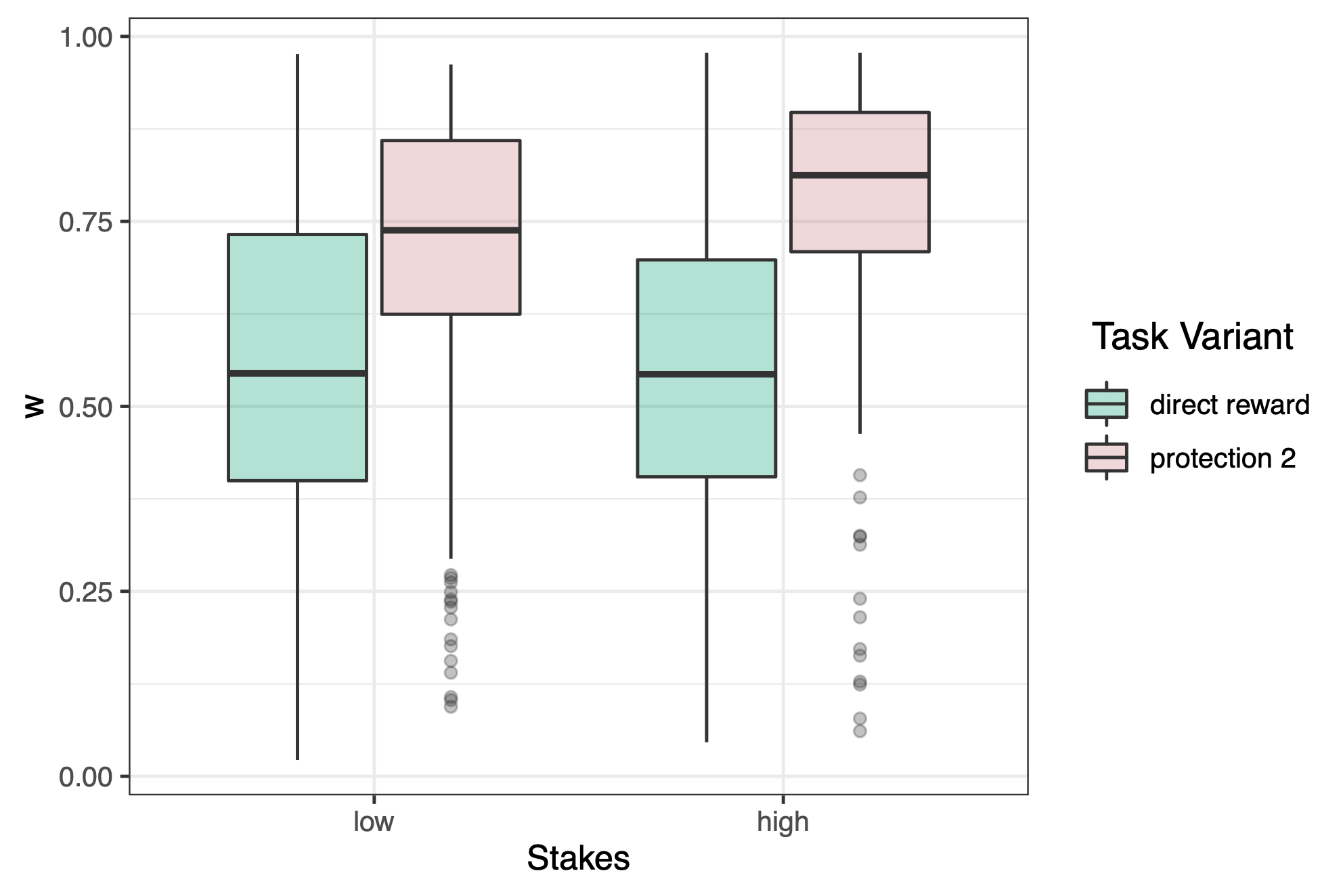

Supplement: S3 Fig — Diverging from Study 1, Study 2 revealed a stakes effect such that model-based weighting (ω) differed between tasks for both high and low stakes, with the protection variant demonstrating more model-based control for both stakes: high stakes wprotection = .70(.21), wdirect.reward = .55(.23), t(199) = 7.40, p < .001, 95% CI [.11, .19], low stakes wprotection = .77(.19), wdirect.reward = .55(.23), t(199) = 11.82, p < .001, 95% CI [.18, .26]. (TIF) [file pcbi.1010805.s003.tif]

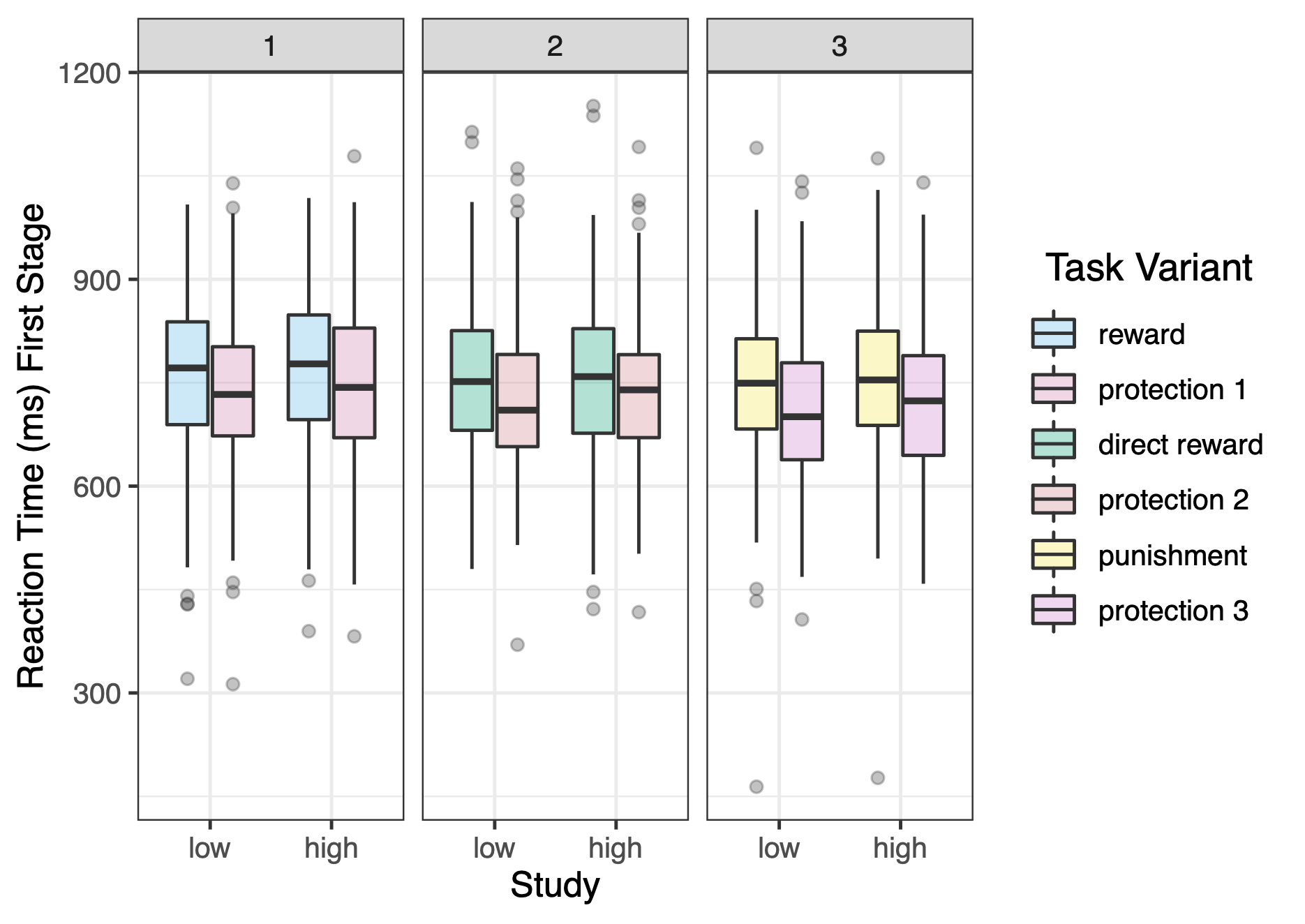

Supplement: S4 Fig — RT for first-stage decisions only differed by stakes for Study 3, such that RTs were slower for high stakes: Estimate = 9.84, SE = 4.75, t = 2.07, p = .039, 95% CI [.52, 19.16], σ2 = 67.23, τ00 = 85.23, R2 = .63. (TIF) [file pcbi.1010805.s004.tif]

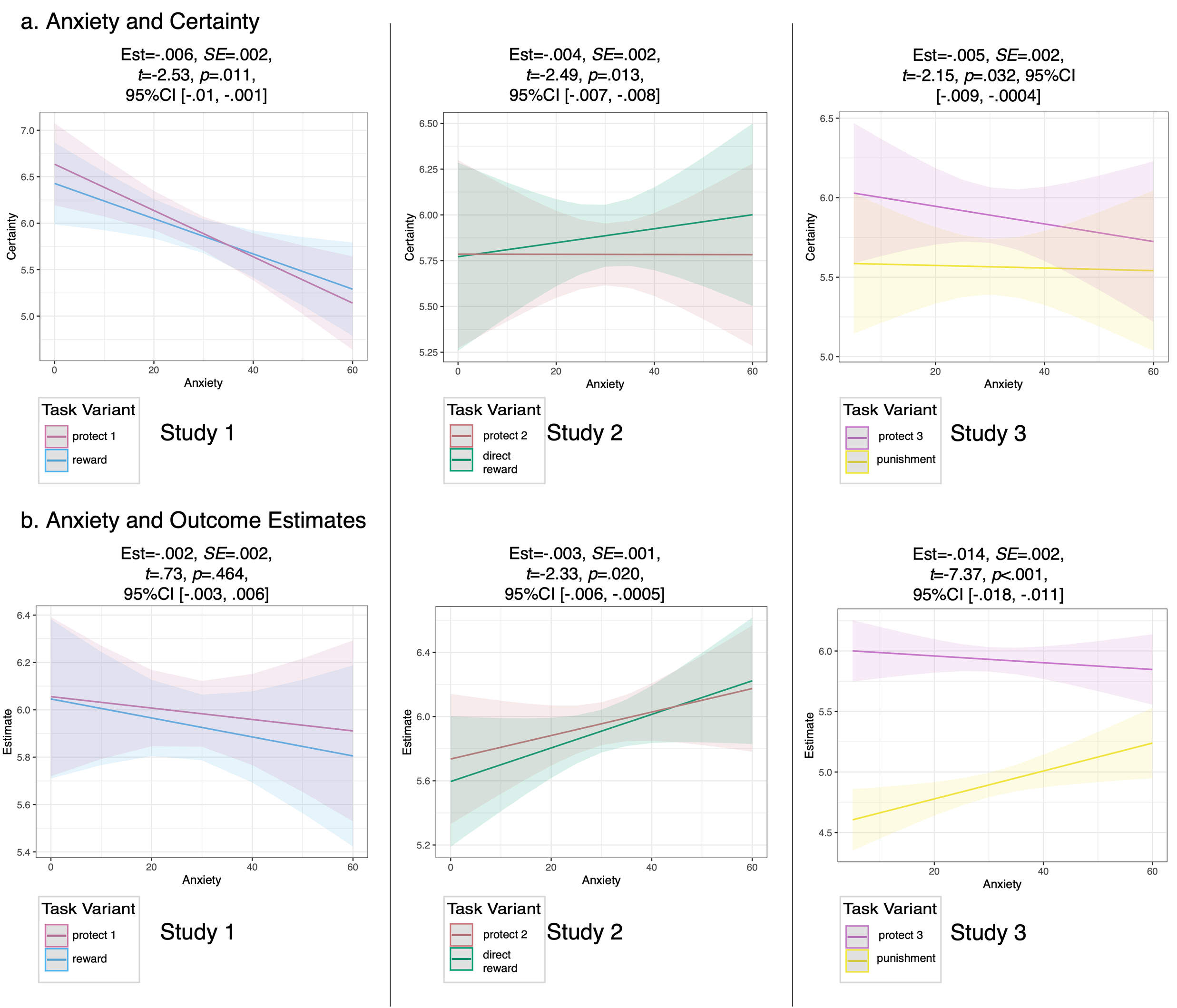

Supplement: S5 Fig — (a) Effects on Certainty of the interaction between anxiety (STAI) and task-variant by Study. (b) Effects on Outcome Estimates of the interaction between anxiety (STAI) and task-variant by Study. (TIF) [file pcbi.1010805.s005.tif]
