# Supplementary material for: Model-based prioritization for acquiring protection
Source: PLoS Comput Biol. 2022 Dec 19;18(12):e1010805. doi: 10.1371/journal.pcbi.1010805 (PMC9810162; doi:10.1371/journal.pcbi.1010805)
Supplement: Attachment — Submitted filename: ResponsetoReviewers_PCOMPBIOL-D-22-00468.pdf [file pcbi.1010805.s008.pdf]

Caltech Humanities and Social Sciences
Computation and Neural Systems

Sarah M. Tashjian, JD, PhD
1200 E. California Blvd.
Pasadena, CA 91125
(626) 395-4065
smtashji@caltech.edu

September 29, 2022

Dr. Ming Bo Cai
Associate Editor
PLOS Computational Biology

Dr. Natalia Komarova
Deputy Editor
PLOS Computational Biology

We sincerely thank the Editors and Reviewers for their time and feedback to our manuscript titled “Model-based prioritization for acquiring protection” [PCOMPBIOL-D-22-00468] for consideration at *PLOS Computational Biology*. In this resubmission we have provided a point-by-point response addressing each Reviewer comment and believe that these changes have improved the manuscript.

We thank you for your time and consideration.

Sincerely,

Sarah M. Tashjian, JD, PhD
Division of Humanities and Social Sciences
California Institute of Technology
smtashji@caltech.edu

Toby Wise, PhD
Division of Humanities and Social Sciences
California Institute of Technology
tobywise@caltech.edu

Dean Mobbs, PhD
Division of Humanities and Social Sciences
California Institute of Technology
dmobbs@caltech.edu

REVIEWER COMMENTS:
Comments to the Authors:
Reviewer #1:

R1.1. I have previously reviewed this manuscript twice at another journal. The authors addressed and incorporated all my points. I have read through the manuscript again and I can't find any other points for the authors to address. I think this manuscript will make an excellent contribution to the field and will be of interest to a broad audience.

A1.1. We appreciate the Reviewer’s continued enthusiasm for this work and for their earlier input that improved this version of the manuscript. Thank you for your time.

Reviewer #2:

R2.1. Tashjian et al. address the question of model-based (as opposed to model-free) control in the context of acquiring protection, as well as in (asymmetric) relation to reward acquisition (i.e., same valence but different context) and harm avoidance (i.e., same context but different valance). To this aim, a well-established two-step task was used across 3 studies using online samples, and the extent of model-based control for protection was found to be consistently higher than reward and punishment tasks. The task/model behavior was further assessed with metacognition and trait anxiety.

This work is very timely and interesting. First, it focused on protection, which was rarely investigated in the literature. Second, it draws direct comparisons regarding the asymmetric relationship between protection and reward/punishment. Overall, the analyses are carefully performed and mostly in support of the key conclusions. The paper is also very well-written. I do have some questions (see below) regarding some of the analyses, and hopefully they help improve the paper.

A2.1. We appreciate the Reviewer's thoughtful input, which we have addressed below.

R2.2. Major points:
(1) Conceptually, the 2x2 distinction between context and valance (Fig 1) is very nice and informative. But to make it really complete, a monetary loss condition should be considered. I am not sure how easily this can be done online, but if not feasible, a comprehensive discussion might be required.

A2.2. We appreciate the desire for a monetary loss condition and perhaps it was not clear that the loss condition (Fig. 1e) is a monetary loss condition. We included a monetary gain (Fig. 1c, 1d) and a protection gain (which mitigates loss, Fig. 1b) as well as a monetary loss condition (Fig. 1e). We clarify this in the introduction "*Protection was equated to reward in that both were appetitive stimuli, but the relevance of acquiring each differed by context such that protection reduced negative outcomes (bonus reduction) whereas reward increased positive outcomes (bonus increase). Punishment was negatively valenced such that it was an aversive stimulus to be avoided and increased negative outcomes (bonus reduction).*" **Subjects received bonus payments as part of the incentive for the task "***The number of second-stage outcomes earned ultimately affected the final result, which was points that contributed to subjects' bonus payments.*" "*Subjects were compensated for their time at a rate of US$9.00 per hour and were entered into a performance-contingent bonus lottery for US$100.00."* **Perhaps the Reviewer is referring to a no reward condition (i.e., positive context, negative valence quadrant of Figure 1a), however this is more akin to a neutral condition and would not be informative with respect to the question of how humans learn about protection in comparison to other appetitive/aversive outcomes.**

R2.3. (2) A few points regarding "Stake"
(2a) It seems that the manipulation of stake was not explicitly introduced in the background, and to be honest, I had to go a bit back and forth to figure out what it meant. So I'd appreciate if this manipulation could be made more evident in either the Intro or the beginning of the Results section.

A2.3. We added discussion of the stakes in the description of the task at the Results section "*On each trial, subjects were first presented with an indication of whether the trial was a high-stakes or low-stakes trial. High-stakes trials were 5x more valuable than low-stakes trials, as indicated by 1 or 5 flames (protection and punishment variants) or 1 or 5 coins (reward variants). The stakes manipulation was designed to test whether model-based control was modulated by incentive and only included a single low- and high-stakes value (1 and 5, respectively).*"

R2.4. (2b) Stake might not only affect the degree of model-based control, but also the exploration-exploitation trade-off. If I got it right, the stake manipulation (x1 vs x5) was presented in a pseudorandomized order. So if a participant had learned the task structure well, she might just want to perform well, irrespective of whether a 1 or 5 will be multiplied. On top of that, if x5 is presented, she may want to maximize the protection given the learned knowledge (ie exploitation), yet if x1 is presented, i.e., the "risk" is low, she may explore the alternative to find out if the reward schedule had changed (exploration). That said, a candidate model that differs in the softmax inverse-temperature shall be considered.

A2.4. We included the stakes manipulation in response to previously published findings regarding increased model-based control as a function of increasing incentives (i.e., stakes) - Kool, W., Gershman, S. J. & Cushman, F. A. Cost-Benefit Arbitration Between Multiple Reinforcement-Learning Systems. *Psychological Science* 28, 1321–1333 (2017).; Patzelt, E. H., Kool, W., Millner, A. J. & Gershman, S. J. Incentives Boost Model-Based Control Across a Range of Severity on Several Psychiatric Constructs. *Biological Psychiatry* 85, 425–433 (2019).

We note there were differences in analytic approach with our work and these prior studies - we used hierarchical Baysian modeling rather than maximum a posteriori model fitting (*Psychological Science* 2017) and fit models with an effect of stakes rather than running separate models for low and high stakes trials separately (*Biological Psychiatry* 2019).

Inverse-temperature interacts multiplicatively with the weighting parameter to determine choice probabilities. This creates a potential non-identifiability issue such that different combinations of parameter values can result in the same likelihood (Gershman, 2016). As such we focused on interpretation of the weighting parameter and did not test multiple candidate inverse-temperature parameters.

R2.5. (3) I find the way to present modeling results first then followed by LME results a bit counter-intuitive. For me, the LME results are model-free/model-agnostic because it does not yet rely on the modeling results; rather, the main effect vs interaction "infers" the MB and MF component. I would first show the LME data, then the modeling/parameter results.

A2.5. We appreciate the Reviewer's suggestion for presenting the LME results first and agree the results could be presented multiple ways. We chose to present the computational results first consistent with existing literature testing two-step tasks and using both computational and LME approaches (e.g., Kool et al., 2017 Psychological Science) and because the computational results were the primary focus of the manuscript.

R2.6. Also, the LME result cannot "[...] validate the computational modeling analyses [...]" (page 6). Instead, the modeling analysis explains the LME findings. To truly validate the modeling results, the authors may consider examining the effect of positive and negative prediction errors (cf. Fig 4, Kool et al., 2017). This way, model-derived decision variables can be connected with the observed behavior, hence validating the modeling results.

A2.6. We changed the word "validate" to "interrogate". We also interrogated prediction error in line with Fig. 4 of Kool et al., 2017 but rather than separately estimating models for low and high stakes and comparing the proportion of trials on which participants chose to repeat the first-stage action as a function of the previous trial outcome, we tested an interaction to examine whether the stakes on each trial moderated the effect of repeating the first-stage action as a function of the prior trial outcome. As indicated in Fig. S2b, this is likely due to the effect of task duration where stakes magnitude becomes relevant at the end of the task but not the beginning. This effect of time is obscured if an interaction is not examined and stakes are compared using separate models.

R2.7. (4) I wonder how omega (MB weight) and alpha (learning rate) are correlated? I am asking because instead of running separate correlations between omega/alpha and the reward rate, a linear regression is more proper: reward rate ~ omega + alpha. This way, the potential correlation between omega and alpha is implicitly considered in the regression model.

A2.7. We now report the correlations between the model-based weighting parameter and learning rate for each study and also supplement the regression for corrected reward rate with a regression accounting for both MB weighting and learning rate.
Study 1: "*ω and α were positively correlated, protection r(199)=.26, p<.001; reward r(199)=.33, p<.001.*"
Study 2: "*ω and α were positively correlated, protection r(199)=.25, p<.001; direct reward r(199)=.38, p<.001.*"
Study 3: "*ω and α were positively correlated for punishment avoidance, r(199)=.15, p=.04, but not protection r(199)=.10, p=.16.*"

Study 1: "*Accounting for correlation between ω and α did not change effect on corrected reward rate, ω Est=.30, SE=.08, t=3.86, p<.001, 95% CI [.15, .46], α Est=.59, SE=.05, t=13.22, p<.001, 95% CI [.51, .68].*"
Study 2: "*Accounting for correlation between ω and α did not change effect on corrected reward rate, ω Est=.16, SE=.06, t=2.81, p=.005, 95% CI [.05, .26], α Est=.54, SE=.03, t=15.74, p<.001, 95% CI [.47, .61].*"
Study 3: "*Accounting for correlation between ω and α revealed no significant association with corrected reward rate, ω Est=.17, SE=.05, t=3.30, p=.001, 95% CI [.06, .27], α Est=.61, SE=.03, t=20.76, p<.001, 95% CI [.55, .67].*"

R2.8. (5) A slightly more motivated description of the models is needed (at the beginning of Page 6). In all the results section, it states that Model 3(or 4) was the best, but it is hard for anyone to know what Model 3 actually is. And, although it seems that the model is well developed, one has to dig into some of the original papers to know the exact model formulae. So a more detailed modeling section would be really beneficial in the Methods section; this is also to make the paper more appropriate for Plos CB. Last, since the authors used Stan for their model fitting, I highly encourage the authors also share their model code (so far I only task code

and data is shared on osf; it is worth also sharing the analysis code). This practice is also in line with the open science policy of Plos CB.

A2.8. For each study results section where we describe the best fitting model, we also list the parameters included in that model "*The best fitting model was Model 3, which included separate model-based weighting (ω) and learning rate (α) parameters for each task variant, as well as eligibility trace (λ), stickiness (π) and inverse-temperature (β) parameters*"**.**
In the Methods under "Reinforcement Learning Models" we describe each model fit and the order in which each model was fit as well as the interpretation of each parameter.
We added the model code to the OSF repository: https://osf.io/4j3qz/.
We added more details of the model (see also A2.13): "*Parameters were specified using non-centered parametrizations, whereby each subject-level parameter ($\theta_{subject}$) is formed by a group-level mean (μ_{group}) and standard deviation (σ_{group}) plus a subject-level offset parameter ($\epsilon_{subject}$):*

$$\theta_{subject} = \mu_{group} + \sigma_{group} \cdot \epsilon_{subject}$$

We used weakly informative prior distributions (normal distributions with mean=0 and standard deviation=1) on each of these parameters and assigned a lower bound of zero for the standard deviations. Subject-level parameters $\theta_{subject}$ were subject to logistic sigmoid (inverse logit) transformations to map them into the range [0, 1]. For the inverse temperature parameter, this was multiplied by 20 to give the range [0, 20]."

R2.9. (6) How initial values of the model was constructed, when the first 20 trials (Study 1 case) were not used in the analysis? It is likely that after 20 trials, participants have already learned at least something of the second-stage values.

A2.9. It is standard practice to exclude a certain number of practice trials from analyses in order to eliminate differences attributable to task orientation (e.g., Kool et al., 2017 doi: 10.1177/0956797617708288; Lockwood et al., 2020 doi: 10.1073/pnas.2010890117). Structuring the task with a random walk requires continuous learning throughout, even if the subjects have already learned something about the structure of the task because the second-stage values change throughout.

R2.10. (7) I am unsure about whether model parameters can be compared (Page 8) if the winning model is not the same. Having additional parameters (Model 4) may take out some variance that omega and alpha could have explained (Model 3) – essentially shifting the marginal distribution from the joint parameter space.

A2.10. Thank you for raising this, we agree. Model 4 was only a slightly better fit than Model 3 for Study 2 (Table S2). We revised the manuscript to report results from Model 3 for all studies (Table S1), which fit well, and moved Model 4 results for Study 2 to supplementals (Fig. S3).

R2.11. (8) Was working memory also measured in addition to metacognition and anxiety? It has been argued that working memory is associated with performance in the two-step task (eg. Collins et al., 2020).

A2.11. We did not measure working memory, but now include this as a limitation and cite Collins et al., 2020. "*We did not examine working memory effects, which have been recently argued to be relevant for performance on two-step tasks.*[37]**"**

Minor points:
R2.12. - At least some of the main statistics should be reported when describing the LME results (on staying probability).

A2.12. We previously did not report these results in the text because parameter estimates and CIs were shown for each task type in Fig 3e. However, we agree with the desire for reported statistics which have now been added to the "Mixed-Effects Models" sections of each study. We also added additional statistics to the "Computational Models" sections of each study.

R2.13. - Page 13, "[…] was performed using weakly informative prior distributions" I guess some transformation was also used (for example, omega, alpha), right? The authors may want to consider following the hBayesDM package paper (Ahn et al. 2017) for model detailed model description.

A2.13. We now include more fulsome details in the manuscript (see also A2.8):
"*Parameters were specified using non-centered parametrizations, whereby each subject-level parameter ($\theta_{subject}$) is formed by a group-level mean (μ_{group}) and standard deviation (σ_{group}) plus a subject-level offset parameter ($\epsilon_{subject}$):*

$$\theta_{subject} = \mu_{group} + \sigma_{group} \cdot \epsilon_{subject}$$

*We used weakly informative prior distributions (normal distributions with mean=0 and standard deviation=1) on each of these parameters and assigned a lower bound of zero for the standard deviations. Subject-level parameters $\theta_{subject}$ were subject to logistic sigmoid (inverse logit) transformations to map them into the range [0, 1]. For the inverse temperature parameter, this was multiplied by 20 to give the range [0, 20].***"**

R2.14. - I am a bit concerned by the learning rate results (Fig2b) – they seem widespread, and many of them are close to 0 or 1. I imagine this would hardly be the case if a hierarchical model was used. So related to my point #5, it would be beneficial to share the Stan code.

A2.14. We added the Stan code to our OSF repository (see also A2.8). High learning rates are fairly typical of this kind of task, and they are generally higher and more widespread than in the traditional probabilistic variant of the two-step task. For example, in Kool et al. (2016, PLOS Comp. Bio.) where a similar type of deterministic transition two-step task was originally reported, the median learning rate is 0.67 with the 25th and 75th percentile being 0.01 and 1.00 respectively, and in Kool et al. (2017, Psych. Science) the mean, 25th and 75th percentiles are 0.5, 0.82 and 1.00 respectively. The learning rates we report have a tighter distribution than reported in these prior studies, with 25th and 75th percentiles further from the bounds of the parameter distribution (as shown in Fig 2b). We believe this is indeed a result of our hierarchical modelling approach.

R2.15. - I am keen to see the actual model comparison results (i.e. WAIC scores) in the results section or SI.

A2.15. We now report the WAIC estimates for each model and each study in Table S2 (copied below). We note that these models are all closely related so the WAIC score differences are small, supporting the use of Model 3 as a comparison across studies (see A2.10).

Table S2. *Watanabe-Akaike Information Criterion (WAIC) scores for each model by study.*

Study	*Model 1*	*Model 2*	*Model 3*	*Model 4*
1	*20877.696*	*20892.668*	*20546.982*	*20612.445*
2	*41399.278*	*41295.218*	*40748.009*	*40673.263*
3	*39849.433*	*39754.564*	*39106.47*	*39171.027*

Note: Model 1: "null model" that did not include an effect of stakes or task variant and accounts for subjects' choices by integrating first-stage value assignment for both model-based and model-free systems. Model 2: included the same first-stage model-based and model-free learning as Model 1 with an additional separate ω and α parameter for the effect of high- and low-stakes trials. Model 3: included the same first-stage model-based and model-free learning as Model 1 with an additional separate ω and α parameter for each task variant. Model 4: included the same first-stage learning and task variant effect as Model 3 with an additional separate ω and α parameter for the effect of high- and low-stakes trials.

Reviewer #3:

R3.1. The authors apply the computational framework of reinforcement learning – which has had enormous success in characterizing reward-based learning & decision-making across many contexts – to a novel context: acquiring "protection", i.e. things that will reduce or prevent future losses. Protection acquisition has been studied as a maladaptive trait in clinical psychology, but not so much as an adaptive computational mechanism under the RL umbrella. In applying RL to protection acquisition, the authors find what to me was a surprising result: People are substantially more model-based when acquiring protection as opposed to just seeking rewards or avoiding punishments. The authors convincingly demonstrate this fact in three pre-registered experiments, and relate this behavior to metacognitive accuracy and anxiety.
I think this is a cool paper, and should be published. Extending RL to the broader range of learning/decision-making contexts that humans experience in their lives – such as protection acquisition – is important & timely, and this paper executes on it well. I applaud their transparency (e.g. with pre-registration) and was convinced of their veracity of their results.

A3.1. We appreciate the Reviewer's enthusiasm for this work and support for publication.

R3.2. I found myself tripped up, however, on some conceptual confusions that I'd love to see addressed. I'm also not totally convinced about the authors' explanation for their results, and am worried about deflationary alternatives. As such, I recommend a substantial R&R.

A3.2. We have addressed the Reviewer's concerns point by point below.

Major points:

R3.3. - Subjects could have just treated your protection task variant as a reward task variant – the protection task (if I understand correctly) is formally equivalent to the direct reward task, except with a negative constant added to the reward function (i.e. you just take whatever reward you got and subtract nine). Am I missing something, or is that right? If I am missing something, then you need to explain the task way more clearly. (You could say it's different because

"shields" are not a primary reward – but then, neither are "fairy coins", so there's really no difference.)

A3.3. This interpretation is somewhat consistent with the task structure. The shields were meant to act as appetitive reward, like the fairy coins. This is the purpose of equating the "valence" of the stimulus that is acquired in the reward and protection variants. The context of the two tasks is different (see Fig. 1a) in that the ultimate outcome in the protection variant is negative and the shields reduce that negative outcome (bonus reduction) whereas the reward variant context is positive and the coins increase positive outcome (bonus increase). We added additional details to clarify this in the introduction "*Protection was equated to reward in that both were appetitive stimuli, but the relevance of acquiring each differed by context such that protection reduced negative outcomes (bonus reduction) whereas reward increased positive outcomes (bonus increase). Punishment was negatively valenced such that it was an aversive stimulus to be avoided and increased negative outcomes (bonus reduction).***"**

R3.4. Assuming I'm not missing something, I think this raises two related concerns. First, is there really reason to think that protection acquisition is conceptually different from the other types of reward learning? I couldn't quite figure out your argument for this in the introduction. Like, you hinged a lot on the difference between reward learning & protection acquisition being that one is in an appetitive context and the other is in an aversive context. Maybe I'm not enough in the appetitive vs aversive literature, but that felt weak to me. Like, in real life, aren't you pretty much encountering both rewards and punishments all the time? (When you build a fence to keep out dangerous animals, you might have built it with your friend and had a good social experience; or taken a break to drink some ice-cold lemonade; or felt a gentle breeze on your face. Is this an aversive context because you're thinking about how to keep out dangerous animals? Or an appetitive context because there's lots of rewards? Given that people treat secondary rewards like money as rewarding/appetitive, why would the protection itself not be treated like an appetitive stimulus? Since the aversive thing is typically not going to happen until far in the future, protection acquisition seems more appetitive than aversive to me!)

A3.4. We appreciate the Reviewer's helpful thoughts and have included a paragraph in the discussion addressing these points: "*In contrast to prior studies that consider safety in terms of punishment avoidance, the current studies aligned protection with reward by making both positively valenced (i.e., more is better). We compared protection with reward to determine whether there was something conceptually different about the way individuals learn for these types of stimuli, or whether protection is simply reward by a different name. Our results suggest the former. We interpret these findings to suggest that, despite similar valence, protection acquisition is conceptually different from other types of reward. The ultimate goal of protection acquisition is to minimize harm, whereas reward acquisition does not explicitly consider harm. During value-based choice, individuals first assigning values to all of the stimuli that can be obtained, and then compare the computed values to select one. In real-world contexts, multiple value-based choices that span appetitive and aversive outcomes may occur simultaneously. For example, perhaps you build a fence with a friend because dangerous mountain lions invade your yard. But while building the fence, you also have positive experiences like sharing time with a friend and drinking a refreshing lemonade. Importantly, the friend and lemonade may be positive but are not protective stimuli. Instead, they are other rewarding social stimuli that co-occur with protection acquisition. Thus, the value-based choice of building a fence versus digging a trench is dissociable from the value-based choice of which friend to invite to help you or the choice of whether to have iced tea or lemonade. The current experimental tasks were designed to*

disentangle appetitive and aversive motivation with respect to the type of outcome faced (reward or loss), as is standard with examinations of appetitive/aversive domains.[27,28] Using both computational modelling and model-agnostic analyses, our findings revealed that protection amplifies contributions from the model-based system when compared with traditional appetitive reward and aversive punishment."

R3.5. And then, in your actual task, the only thing that differs between the two is a constant in the reward function. Is that really enough to call it a fundamentally different context? Clearly that manipulation did actually change people's behavior (although see the next paragraph) – am I just missing how much people really treat "positive reward function context" vs "negative reward function context" as fundamentally distinct? I'm totally open to being convinced of that, but I wanted an argument for it more explicitly (addressing these issues).

A3.5. See A3.11. additions to the introduction to clarify the differences among task variants and conceptualization of protection. We note that if people were treating "positive reward function context" versus "negative reward function context" as fundamentally distinct without taking into account context *and* valence, we should also see differences between the purely appetitive reward and aversive punishment variants.

R3.6. - The second, related concern is that there's a boring explanation for why people are more model-based in the protection variant. My worry was that it's just something like: The shields variant is just weirder or less natural for people, and puts them on "high alert" in a way. Like, getting a thing which then prevents another thing feels like more cognitive steps to me somehow than the other variants (even the one with the sacks? I didn't really get that one anyway – how did the sacks differ from coins?).

A3.6. We would expect if the protection variant was "weirder" there would be a difference in learning rate as individuals were taking more cognitive steps to sort out the contingencies. We agree that it isn't perfect to have an indirect delivery of punishment that is mitigated by protection, but that is the way protection functions in the real world (it moderates punishment). To mimic this intermediate moderation with reward, we created an indirect reward variant where sacks were used as an intermediate step to "carry the coins". We agree that the sacks more closely approximate reward which is why we conducted the conceptual replication with the direct reward variant excluding sacks.

R3.7. You might come back and say: That's exactly our hypothesis! That the "valence-context asymmetry" inherent in protection acquisition necessitates more model-based control. But this argument doesn't sit well with me. First, the way you put it in the text is that valence-context asymmetries may require more "flexible action policies", and you hint that this has something to do with the fact that non-protection cases have more "predictable environments". But the tasks are formally equivalent except for a constant in the reward function. Protection tasks don't seem any more unpredictable to me; there's no actual, formal need for more flexibility in the protection variant than the other variants. (Another way to put this is that the model-based advantage – i.e. how much more reward a model-based algorithm got on average vs a model-free algorithm – would not be higher for the protection variant vs. the other variants of your task.)

A3.7. We based our argument on valence-context asymmetries on prior work related to the comparison between reward and punishment (Alves, Koch, &

Unkelbach, 2017 Trends in Cognitive Sciences; "There are robust asymmetries in the processing of positive and negative information at virtually all levels of human information processing.... Negative information draws more attention, leads to stronger neurological reactions, and is recognized more accurately...we propose that some valence asymmetries might not be caused by internal affective or motivational forces but <u>may originate in the structure of the information itself</u>. Specifically, positive and negative information generally differ regarding a crucial property, namely, similarity. Positive information is more similar to other positive information, compared to negative information's similarity to other negative information. Our explanation for positive information's higher similarity builds on the well-documented assumption that valence is a function of attribute extremity. ... a positive range is located toward the middle of a given attribute dimension and is surrounded by two negative ranges toward the two ends of the dimension. Thereby, positivity is non-extreme."**). If this theoretical comparison were only justifiable when tasks are not formally equivalent, as the Reviewer suggests, this logic would apply to comparisons of reward and punishment when tasks are equivalent. However, there is support for different contributions of decision policies in reward and threat contexts (e.g., Worbe et al., 2016 Molecular Psychiatry; Voon et al., 2015 Translational Psychiatry; Park et al., 2017 PLOS ONE). The key here is that there may be different contributions of model-based and model-free control when *not required* by the task *because of the nature of the outcome*.**

We also support our assertion that protection acquisition may require more model-based control than reward acquisition and punishment avoidance based on interactions between affect and action (e.g., Guitart-Masip et al., 2012 Neuroimage; "Decision-making invokes two fundamental axes of control: affect or valence, spanning reward and punishment, and effect or action, spanning invigoration and inhibition... One abundant source of sub-optimalities is the substantial interdependence of two logically independent axes of behavioral control: a valence axis running from reward to punishment, and an action axis running from vigor to inhibition. Pavlovian responses associated with predictions of reward usually entail vigorous active approach and engagement, irrespective of the instrumental validity of these actions. Equally, Pavlovian responses to (at least distal possible) punishments are generally associated with behavioral inhibition.... Pavlovian value expectations can disrupt instrumental performance, with anticipation of punishment impairing active go responses. However, the studies concerned considered steady-state behavior in a stable world, and did not examine learning. This is a critical omission, since the interaction between action and valence could boost, or indeed prevent learning altogether."**). Protection here tests the interaction between action and valence by requiring approach toward protective stimuli during anticipation of punishment.**

Other related work supports our assertion that investigation of valence-contexts asymmetries is warranted, but take different approaches that do not specifically consider the natural variation in protection compared with reward and threat (e.g., Gaillard et al., 2019 Brain and Behavior; Hu, Padmala, & Pessoa, 2013 Neuropsychologia; Penner et al., 2022 Cognitive, Affective, & Behavioral Neuroscience).

R3.8. Moreover, if you think I'm right that the increase in model-basedness is due to some kind of "high alert / weirdness" thing, then that's not at all specific to protection. Anything I did to make the task weirder or less natural would cause it. For instance, imagine I told people that a random varying amount (positive or negative) would be added to their bonus each trial. That would not induce a consistent valence-context asymmetry in the same way – would it still make

people equally model-based? I kinda think it would. Or imagine that I did a weird variant where fewer coins meant they received more bonus money at the end.. I'd make the same prediction there.

You might say, “Fine, the cause – inducing high-alert-ness – is extremely unspecific to protection, but as a matter of fact it *does* apply to protection cases, so it *will* actually make people more model-based in those cases”. That’s fine – but then I think the framing needs to be different. If that’s what you think is going on, you can’t frame it as something remotely unique to protection cases, and you’d have to really emphasize that this just happens to be a feature of protection that is making people more model-based. (Also, if this is what’s going on, it would make me worry about generalizability – like, do protection cases actually put people on high alert in real life?)

A3.8. We appreciate the intrigue of this conjecture, but if it were that “weirdness” were driving the alertness of the model-based control contribution, then we should see this similar effect difference when the reward variant involves collecting the sacks as opposed to the coins. However, MB weighting actually increased for the direct reward variant suggesting “weirdness” is not driving this effect. Direct testing of underlying mechanisms for our observed effects would be most appropriate for follow-up studies.

R3.9. In contrast, if you disagree with me that my “high alert / weirdness” hypothesis is what’s explaining the difference between conditions, then I think you need to:

(a) make an argument for why valence-context asymmetry (i.e. adding a constant to the reward function) actually requires more flexibility / model-basedness, or identify a different reason why a valence-context asymmetry would engender more model-based-ness;
(b) give some evidence (or reason to think) that “high alert / weirdness” is not explaining the effect.

If you disagree with my “high alert / weirdness” hypothesis, the strongest thing I think you could do for the paper would be to propose a compelling alternative and run another experiment that adjudicates between them. But I’m not at all requiring that for revision, and I think it’s totally possible that you could convince me by doing some analyses of the existing data you have. For example: Could the stakes conditions help inform these questions somehow? Like, I know higher stakes should also put people on higher alert.. Did you find a stakes effect in Studies 2-3? I couldn’t really tell from your description of the results.

A3.9. See above A3.8. response to the “weirdness” hypothesis. We did not find a stakes effects in studies 1 and 3. The model including stakes was a slightly better fit for Study 2, which was driven by the direct reward variant and discussed in the results: “Computational Model 4 was the best fitting model for Study 2, which included separate ω and α parameters for task variant and stakes. ω was higher for the protection variant compared to the direct reward variant, consistent with Study 1 (Fig. 2a). Diverging from Study 1, ω differed between tasks for both high- and low-stakes trials, with the protection variant demonstrating more model-based control for both stakes (Fig. S3).” “Stakes interacted with the model-based component, which was driven by the direct reward variant (Fig. S2c). No significant stakes interaction was present for the model-free component.”

R3.10. Another analysis you could run that would be informative for this question is to look at whether people stay consistently more model-based in the protection variant throughout the experiment. If it's really some kind of "oh this is weird, I should be more careful" thing going on, then I'd predict you should only really find the increased model-based-ness in the first half of the experiment and not the second.

(You might turn to the fact that people are no slower – in fact, they're faster? – in the protection variant, as evidence that people don't find the task weirder. But I'm not really convinced by that. It fits with the "high alert" hypothesis in my head.)

A3.10. We appreciate the Reviewer's question, but splitting the data over time reduces the amount of data used for model estimation making the weighting parameter less accurate and there are other factors confounded with time that influence the weighting parameter including how familiar people become with the task structure and the point in time when the reward distribution shifts according to the random walk. Additionally, because this analysis would be under-powered, whether it fits or doesn't fit with the Reviewer's interpretation would be minimally informative because the result could be spurious in either direction. For these reasons we don't think this would actually benefit the interpretation of results here.

To attempt to respond to the Reviewer's latter point about RT, we tested whether trial number interacted with task type to predict RT. Across all task variants, RT reduced over the duration of the task. If results were due to high alertness on the protection variant, we should see that RT speeds up for non-protection, but stays consistent for protection. That is not what we find.

Study 1:
Trial – Est=-.09, *SE*=.05, *t*=-2.07, p=.04
Trial * task type – Est=.03, *SE*=.06, *t*=.46, p=.64
Trial, protection only – Est=-.52, *SE*=.06, *t*=-9.32, p<.001

Study 2:
Trial – Est=-.04, *SE*=.02, *t*=-2.43, p=.02
Trial * task type – Est=-.007, *SE*=.02, *t*=-.33, p=.74
Trial, protection only – Est=-.31, *SE*=.02, *t*=-14.82, p<.001

Study 3:
Trial – Est=-.04, *SE*=.01, *t*=-3.05, p=.002
Trial * task type – Est=-.03, *SE*=.02, *t*=-1.74, p=.08
Trial, protection only – Est=-.28, *SE*=.02, *t*=-13.79, p<.001

R3.11. Just to add one more thing to this train of thought: Even if there's no meaningful formal difference between protection acquisition cases and other types of reward learning, maybe there's still a *psychological* difference? Like, people categorize it differently in their heads? Is that what you think is going on? For instance, I'd be really curious to see a version of your protection variant where, instead of framing it as protection, you frame it as just subtracting a constant amount from your reward every time. Do you predict that people would still be more model-based there? If yes, that would be a very strong test of your hypothesis. If no, then is it really about a valence-context asymmetry, or is it something else (and what is it)?

Anyway, there's a ton of thoughts in there – as you can see, I found myself a bit jumbled on these questions. If you can find some way to convincingly clarify these issues, I would be enthusiastic about this paper being published :).

A3.11. We appreciate the nuanced interpretations and questions raised by our manuscript. One issue with the Reviewer's suggestion to modify the protection variant as a loss is that this would, in effect, mimic the punishment avoidance variant. We have already answered the question with respect to whether framing of subtracting a constant from reward would shift model-based weighting - subjects are not more model-based for punishment compared to reward.

In response to the Reviewer's note that these issues are not clear and in specific response to A3.5., we reworked the framing in the introduction "*Protective decisions are distinct but retain superficial similarities to both reward- and punishment-motivated decisions. Protection is positively-valenced, similar to reward but unlike punishment. Protection exists in a negative context, similar to punishment but unlike reward (Fig. 1a). Additionally, protection is distinct in the degree to which valence and behavior are aligned, which has consequences for learning.[11,12,13] Negatively-valenced stimuli like punishment typically elicit avoidance behaviors, whereas positively-valenced stimuli like reward elicit approach behaviors.[14] In this study, subjects were incentivized to actively seek out the maximum protection available as opposed to avoid the highest punishment. This aligns with traditional definitions of approach motivation as the energization of behavior toward a positive stimulus.[15] Prior studies of decision control typically exploit the conventional coupling of valence and context (i.e., positively-valenced outcomes in an appetitive context or negatively-valenced outcomes in an aversive context). This perspective does not sufficiently identify how decision control systems contribute to acquiring protection because protection decisions involve asymmetric valence and context.*"

We also acknowledge that there is support for competing hypothesis with respect to model-based contributions if the context-valence asymmetry of protection is indeed more "weird" or complex: "*Protective decisions are also largely absent from traditional conceptualizations of safety, which consider the cessation of punishment but do not consider circumstances in which punishment is reduced through the conferral of positive protective stimuli.[1] Thus, it remains an open question whether the decision control systems for protection differ from reward, with which it shares a positive valence, or from punishment, with which it shares a negative context. It is possible that the context-valence asymmetry of protection has no effect on the computational decision structure when compared with these traditional stimuli. In prior work, reward acquisition and punishment avoidance elicit similar weighting of model-based control, suggesting that there may be some common substrate for reinforcement learning irrespective of stimulus properties.[11,16] However, reward and punishment are valence-context congruent. This valence-context symmetry[17] could favor model-free control as a result of less complex contingency learning.[5,13] Support for this hypothesis is evident in predictable environments where reward learning engages goal-directed control early on, but cedes to habitual control as an efficiency.[18] By contrast, amplification of prospective model-based control can aid development of accurate and flexible action policies,[11], which may facilitate response to hierarchically-organized motivational demands (approach toward protection with a broader goal to avoid punishment). Thus, the valence-context asymmetry of protection may bias toward greater prospective model-based control than both comparison stimuli. Alternatively, it is possible that valence-context asymmetry increases perceived difficulty resulting in increased model-free contributions as a form of learned helplessness.[10]*"

Some smaller things:

R3.12.- I was very confused by the task at first read (and still am a bit confused, even after digging into the methods section). I couldn't tell whether it was always the same amount of flames on each trial (assuming the same stakes condition), or whether that varied randomly across trials. I couldn't tell what the sacks did. Don't make the reader dig into methods section (or, God forbid, the Supplement) to understand these things.. I think you should do some work to make the task description way clearer.

A3.12. We include a more comprehensive task description at the start of the results
"*In each pre-registered study, a balance between model-free and model-based control was assessed using two variants of a two-step reinforcement learning task. Each study included a protection acquisition variant and either a reward acquisition (Studies 1 and 2) or punishment avoidance comparison (Study 3). During each task, subjects made sequential decisions that navigated them through two "stages" defined by different stimuli. Subjects were told at the outset that they were traveling through a fictious forest. On each trial, subjects were first presented with an indication of whether the trial was a high-stakes or low-stakes trial. High-stakes trials were 5x more valuable than low-stakes trials, as indicated by 1 or 5 flames (protection and punishment variants) or 1 or 5 coins (reward variants). The stakes manipulation was designed to test whether model-based control was modulated by incentive and only included a single low- and high-stakes value (1 and 5, respectively). After the stakes depiction, subjects were shown one of two first-stage states. Each first-stage state included 2 dwellings and subjects chose one dwelling to visit (left or right). In the protection variant dwellings were trees, and in the reward and punishment variants dwellings were houses. First-stage dwellings were randomly presented in 2 equivalent states such that dwellings remained in their pairs throughout but the position of each dwelling (left versus right) was counterbalanced across trials. In total, 4 total dwellings were available for each task variant. In each of the first-stage states, one dwelling led to one creature and the second dwelling led to a different creature (2 total creatures), creating an implicit equivalence across first-stage states. Dwelling-creature pairings remained constant (deterministic transitions). Each second-stage creature was associated with a fluctuating outcome probability. Across all protection task variants, the second-stage outcomes were protection stimuli that reduced losses (shields to protect against the dragon flames). In the Study 1 reward task variant, the second-stage outcomes were reward stimuli that increased gains (sacks to carry the fairy coins out of the forest). In the Study 2 direct reward task variant, the second-stage outcomes were directly delivered as reward stimuli (fairy coins) that increased overall gains. In the Study 3 punishment task variant, the second-stage outcomes were directly delivered as punishment stimuli (dragon flames) that increased overall losses. Second-stage probabilities changed slowly over time, requiring continuous learning in order select the appropriate first-stage state that led to the second-stage creature that provided the most optimized outcome. At the final frame, second-stage outcomes (shields, sacks, coins, flames) were multiplied by the initial stakes to compute an overall point result for that trial, which affected the subject's bonus payment.***"**

R3.13. - I had a couple concerns about the model comparison method. I'd never heard of the WAIC before. At first I assumed it was just another criterion like AIC, BIC, or DIC which tries to correct for overfitting with the raw number of parameters (which is a really bad way to do it). But then I looked into it and realized it's more complex than that in a way I didn't exactly understand? Anyway, I think it would be really helpful to explain & justify the use of the WAIC here, for folks like me who don't know it :).

Also, I was always taught that the best ways to do model comparison were to either use the random effects method from Stephan et al (i.e. estimate the model evidence using the Hessian matrix, treat model parameters as random effects across subjects, and compute exceedance probabilities; e.g. https://www.sciencedirect.com/science/article/abs/pii/S1053811909002638, http://www.cns.nyu.edu/~daw/d10.pdf, https://github.com/sjgershm/mfit), or to just do normal cross-validation. Is there a reason to do an asymptotic approximation like WAIC here, instead of one of those methods?

A3.13. WAIC is a well-recognized method of model comparison for Bayesian models and is designed to use the full posterior distribution over parameter estimates rather than using point parameter estimates. We also agree that true cross-validation would also represent an effective method for model comparison, however this would require that we can split the data into relatively independent fitting and evaluation sets. In tasks like this one this may not be a safe assumption as subjects' strategies may be subject to subtle changes throughout the task, for example learning rates may change throughout the task in response to local volatility and estimation uncertainty. As a result, we believe the WAIC provides the most appropriate way to obtain a measure of model fit that accounts for complexity.

We added additional language on WAIC estimations and make reference to the 2014 publication by Gelman, Hwang, and Vehtari for those who are interested in reading more. *"Watanabe-Akaike Information Criterion (WAIC) scores were used as a complexity-sensitive index of model fit to determine the best model for each study. WAIC estimates expected out-of-sample-prediction error using a bias-corrected adjustment of within-sample error, similar to Akaike Information Criterion (AIC) and Deviance Information Criterion (DIC)[22]. In contrast to AIC and DIC, WAIC averages over the posterior distribution rather than conditioning on a point estimate, which is why WAIC was selected as the index of model fit."*

R3.14. - In the fourth paragraph, you write: "In prior work, reward acquisition and punishment avoidance elicit similar weighting of model-based control." But then later, you write: "… in line with prior work showing aversive contexts decrease model-free contributions to reward learning." Are those contradictory, or am I missing something?

A3.14. The study we referred to as "*prior work showing aversive contexts decrease model-free contributions to reward learning***" did not compare reward and punishment learning but rather learning when there was a neutral background images (e.g., paper clips) versus negative background images (e.g., IAPS images). In both cases, the outcome was the same (money) but the background prime was either neutral or negative. Only MF control was affected in that study such that negative background images increased MF learning when subjects had to avoid punishments but decreased MF learning when subjects had to approach rewards. We edited that sentence for clarity: "***We hypothesized that model-based contributions for protection would also be higher compared to punishment avoidance given the potential for combined contributions of appetitive and aversive motivations for protection (Study 3). We examined effects of incentives (high versus low stakes) to determine whether differences in model-based control were modulated by incentive.[19,21] We hypothesized that incentive sensitivity would be higher for reward given lower value thresholds for protection and punishment. Lastly, we examined metacognitive and predictive accuracy on each task to*

determine how awareness of task performance related to model-based control. We hypothesized increased model-based control would be associated with better metacognitive accuracy across all stimuli."

R3.15. - For the metacognitive analysis: Don't you need to do some really fancy stuff to correctly analyze metacognitive data? E.g. see Fleming & Lau (2014), "How to measure metacognition", https://www.frontiersin.org/articles/10.3389/fnhum.2014.00443/full. They say you have to estimate an ROC curve, etc etc. I know the metacognitive stuff is not the main point of your analysis, so this may not be worth it, but it's worth considering (and, if you don't do it, justifying why you don't do it).

A3.15. In accordance with Fleming & Lau (2014), our question pertains to metacognitive and predictive bias, or the difference in subjective confidence despite task performance. This is a better measure of awareness, as opposed to accuracy which is a measure of sensitivity. Additionally, we are using within subject measures to assess differences by task variant, which takes into account baseline responding attributable to individual differences in personality rather than metacognitive ability. We clarify the measure of bias in the introduction and "*Lastly, we examined metacognitive and predictive bias on each task to determine how difference in subjective confidence and task performance monitoring related to model-based control and anxiety. We hypothesized increased model-based control would be associated with reduced metacognitive bias (improved correspondence between confidence and performance) across all stimuli.*" **We have updated the manuscript to refer to metacognitive and predictive bias throughout. Additionally, as the Reviewer noted this is not our main analysis, but rather an additional measure of the different ways individuals process protection compared to other similar stimuli.**

R3.16. - I didn't understand the anxiety analysis. Are you saying you found a three-way interaction between study1/2 vs 3, model-based-ness, and STAI score? How should we interpret that? Are the main effects significant within study? When I first read the result, I thought you meant that high-anxiety people had a monotonic ordering of model-basedness in reward seeking > protection acquisition > punishment avoidance.. Is that right, or is it some kind of crossover interaction that I'm not understanding? It took me forever to figure out how that result mapped onto the graph in Figure 4. I think you should graph that by study separately (as you do in the other figures); I initially just read the graph as random noise differing b/w studies. I still don't really know how to interpret that graph. Also, the primary analysis I was expecting was a first-order correlation b/w anxiety and model-based-ness (or model-free-ness) in protection acquisition, ignoring the other conditions. Do you find that? If not, what are the implications?

A3.16. We clarify that the analysis assessed the within-subject difference in model-based weighting (*w*) within each study (i.e., to what extent is protection higher than non-protection?) interacted with study type (i.e., protection versus reward, protection versus direct reward, protection versus punishment) predicting STAI. Results: "*Differences in deployment of model-based control (ω-difference score calculated as non-protection variant subtracted from the protection acquisition variant for each study) were associated with anxiety such that individuals with higher scores on the STAI demonstrated greater model-based weighting for reward acquisition compared with protection acquisition, but greater model-based weighting for protection acquisition compared with punishment avoidance: study by ω-difference interaction Estimate=5.40, SE=2.21, t=2.45, p=.015, 95% CI [1.07, 9.74], R^2=.02 (Fig. 4).*" **Discussion:** "*Individual differences in trait anxiety were associated with degree of model-based control deployed to acquire*

protection, offering a potential mechanistic explanation for differences in safety decisions previously documented in anxious individuals.[31] *For individuals with higher anxiety, model-based control for protection was decreased compared with reward. In a separate sample, model-based control was elevated for protection compared with punishment. This increase in model-based control depending on valence-context interactions also supports our assertion that protection acquisition is distinct from purely aversive punishment and appetitive reward."* **Fig. 4. Legend:** "*Anxiety and model-based weighting (ω) estimated separately for each Study.*"

It is not possible to assess monotonic ordering within-subject because subjects only completed 2 of the 4 variants depending on which study they participated in. We did not test the correlation between protection acquisition model-based weighting and anxiety only because we wanted to use the baseline of the non-protection weighting to indicate how disparate the two weighting parameters were as a within subject measure of protection MB bias rather than a between subject measure of model-baseness more generally.
