# Supplementary material for: Model-based prioritization for acquiring protection
Source: PLoS Comput Biol. 2022 Dec 19;18(12):e1010805. doi: 10.1371/journal.pcbi.1010805 (PMC9810162; doi:10.1371/journal.pcbi.1010805)
Supplement: Attachment — Submitted filename: ResponsetoReviewers_PCOMPBIOL-D-22-00468R1.pdf [file pcbi.1010805.s009.pdf]

Caltech Humanities and Social Sciences
Computation and Neural Systems

Sarah M. Tashjian, JD, PhD
1200 E. California Blvd.
Pasadena, CA 91125
(626) 395-4065
smtashji@caltech.edu

December 1, 2022

Dr. Ming Bo Cai
Associate Editor
PLOS Computational Biology

Dr. Natalia Komarova
Deputy Editor
PLOS Computational Biology

We sincerely thank the Editors and Reviewers for their time and feedback to our manuscript titled “Model-based prioritization for acquiring protection” [PCOMPBIOL-D-22-00468R1] for publication at *PLOS Computational Biology*. In this letter we have addressed the remaining points provided by Reviewer 2.

Sincerely,

Sarah M. Tashjian, JD, PhD
Division of Humanities and Social Sciences
California Institute of Technology
smtashji@caltech.edu

Toby Wise, PhD
Division of Humanities and Social Sciences
California Institute of Technology
tobywise@caltech.edu

Dean Mobbs, PhD
Division of Humanities and Social Sciences
California Institute of Technology
dmobbs@caltech.edu

EDITOR NOTE:
The reviewers appreciated the attention to an important topic. Based on the reviews, we are likely to accept this manuscript for publication, providing that you modify the manuscript according to the review recommendations.

Please consider the additional comments from Reviewer 2. The editors will check the revision without sending out for another review.

REVIEWER COMMENTS:

Reviewer #2:

(1) In R2.4 “[…] On top of that, if x5 is presented, she may want to maximize the protection given the learned knowledge (ie exploitation), yet if x1 is presented, i.e., the “risk” is low, she may explore the alternative to find out if the reward schedule had changed (exploration)”, the authors did not directly answer this question. Testing inverse-temperature resulting non-identifiability could indeed be an issue, but this does not mean the stake did not affect the

potential exploration-exploitation trade-off – something that should be at least briefly discussed.

We added discussion to the limitations regarding the potential of a stakes-related explore-exploit tradeoff on page 12 "Although we based our paradigm development on widely-used and validated reinforcement-learning tasks, we only replicated the stakes effect observed in prior work in Study 2.[21] The model accounting for both task variant and stakes fit best for Study 2, but the WAIC score for the more complex model was only .18% different from the simpler model, thus we used Model 3 to compare across studies. Despite prior work identifying higher exploit behavior under high-stakes,[19] we did not test an inverse-temperature difference by stakes considering the lack of stakes effect on model-based weighting and the potential for non-identifiability given inverse-temperature interacts multiplicatively with the weighting parameter.[39]"

(2) In R2.5, I slightly disagree that the authors chose to follow the original way of presentation (cf. Kool et al 2017). Speculatively, even when the original authors are using the same paradigm again, they may also revise the way of presenting results.
Here, I respect the authors' decision. But it helps to explicitly mention that it is intended to follow Kool et al 2017 closely, though the way of presenting is somewhat counterintuitive.

We now add explicit mention that our presentation of results follows Kool et al. 2017 on page 6. "To interrogate the computational modeling analyses, we used mixed-effects logistic regressions. We present computational results first, followed by mixed-effects regression results consistent with prior work[19] and because the computational results are of primary focus here. Regression was used to examine choice behavior as a function of the outcome on the previous trial and similarity in first-stage state."
